# Regrowth Rate of a Cardiac Myxoma After Percutaneous Aspiration

**DOI:** 10.1016/j.jaccas.2024.102850

**Published:** 2025-01-02

**Authors:** Zach Rozenbaum, Eric Gnall

**Affiliations:** a Department of Cardiology, Tulane University, New Orleans, Louisiana, USA; b Department of Cardiology, Lankenau Medical Center, Wynnewood, Pennsylvania, USA

**Keywords:** echocardiography, growth rate, myxoma, percutaneous aspiration, regrowth rate, shortness of breath, treatment

## Abstract

Cardiac myxomas are typically treated surgically; however, the operative mortality and recurrence rates are not negligible. In the current report we describe a case of repeat percutaneous aspiration of a right atrial myxoma. The report supports feasibility of the procedure, provides the asymptomatic timeframe after debulking, and the regrowth rate.

## Graphical Abstract

**Figure:**
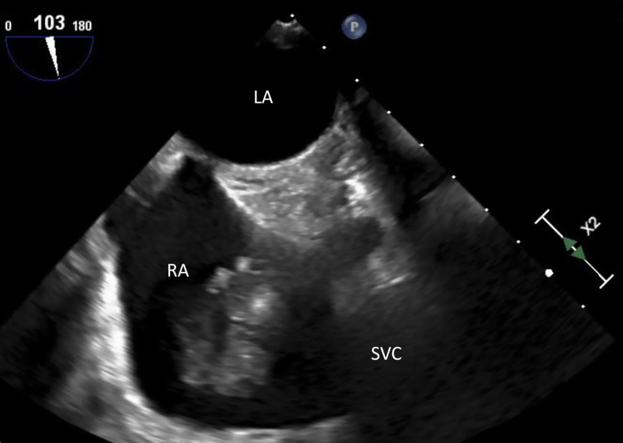

## History of Presentation

A 70-year-old woman presented to the emergency department with insidious progressive nonexertional shortness of breath. She also complained of occasional nonpositional dizziness and an episode of syncope. During her workup in the emergency department, she underwent transthoracic echocardiography and was found to have a large right atrial mass. She was transferred to a tertiary center for further evaluation.

**Take-Home Messages**

• Percutaneous aspiration for right atrial myxoma is feasible for nonsurgical candidates.
• The regrowth rate was comparable to that of in situ tumors.
• A successfully debulked myxoma may be a mid-term solution in selected patients.

## Past Medical History

Past medical history included hypertension, hyperlipidemia, anxiety, asthma/chronic obstructive pulmonary disease, hypothyroidism, spinal stenosis (ambulates with a walker due to chronic backpain), cutaneous lupus, and morbid obesity.

## Differential Diagnosis

Differential diagnosis includes a thrombus and neoplasms—primary or secondary tumors. The more common neoplasms in the right atrium include myxoma, lymphoma, sarcoma, metastasis, and lipoma.^1^

## Investigations

**Figure 1:**
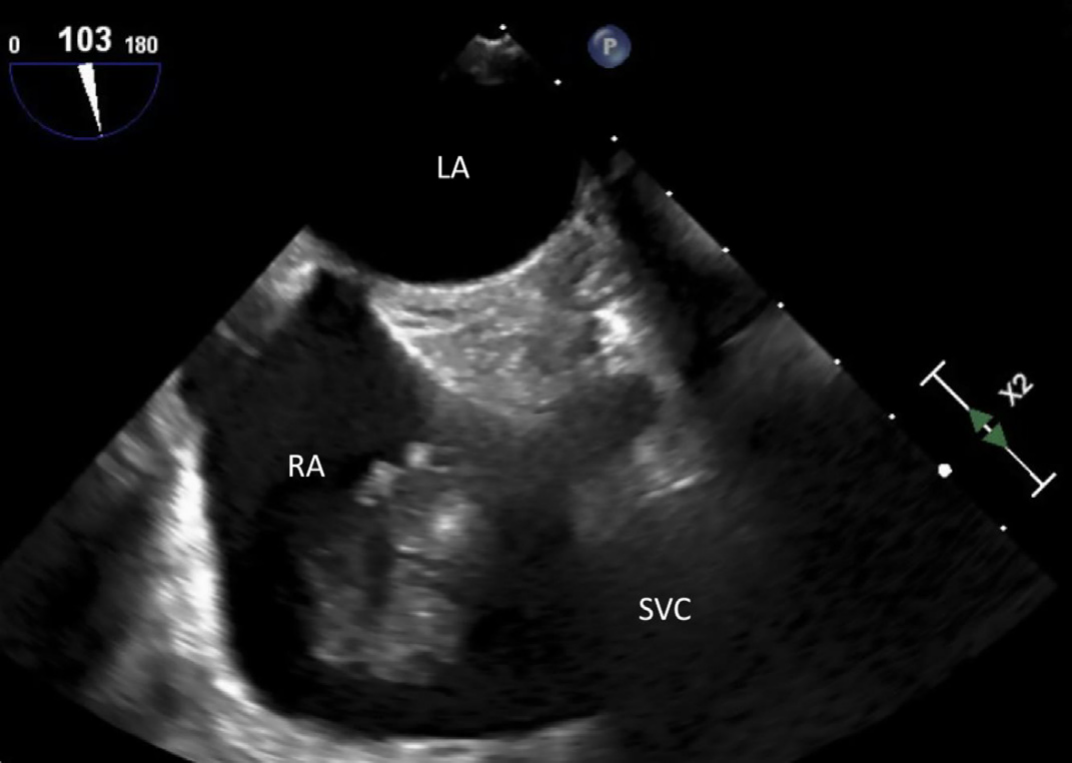
Transesophageal Echocardiography Showing a Right Atrial Mass LA = left atrium; RA = right atrium; SVC = superior vena cava.

**Figure 2:**
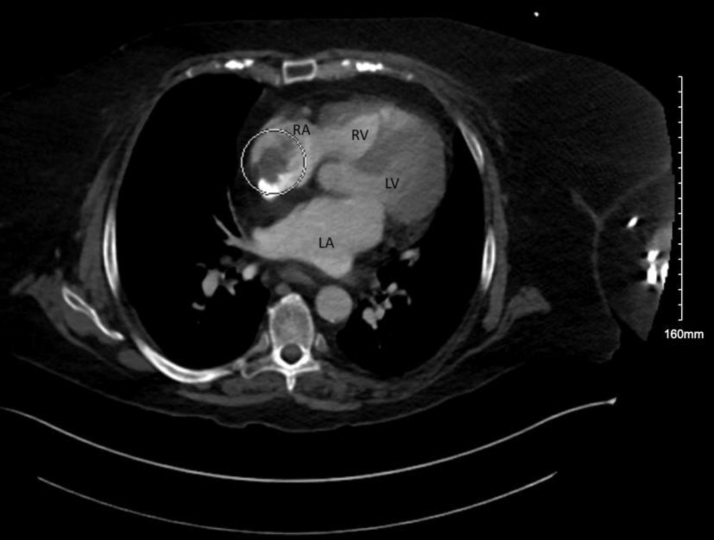
Computed Tomography Showing a Right Atrial Mass LA = left atrium; LV = left ventricle; RA = right atrium; SVC = superior vena cava.

There were no constitutional symptoms on review of system and no remarkable findings in the physical examination. Electrocardiogram and telemetry showed no signs of atrial fibrillation or other atrial arrhythmias. There were no signs of active infection, antiphospholipid antibodies were negative, and there was no eosinophilia. Transesophageal echocardiogram showed (Figure 1) a 2.3 × 3.6-cm multilobulated mass in the right atrium attached to the superior vena cava–right atrial junction. Computed tomography with contrast showed (Figure 2) a nonenhancing mass attached to the vena cava and extending into the right atrium, with no additional relevant systemic foci.

## Management

A multidisciplinary discussion was performed. The patient did not have intracardiac foreign bodies or known atrial fibrillation. The most likely diagnoses were thrombus and myxoma. There were already symptoms consistent with obstruction of flow and she was at a high risk of cardiac surgery due to chronic obstructive pulmonary disease, morbid obesity, and spinal stenosis, which may hinder surgical recovery. Therefore, percutaneous aspiration was pursued.

**Figure 3:**
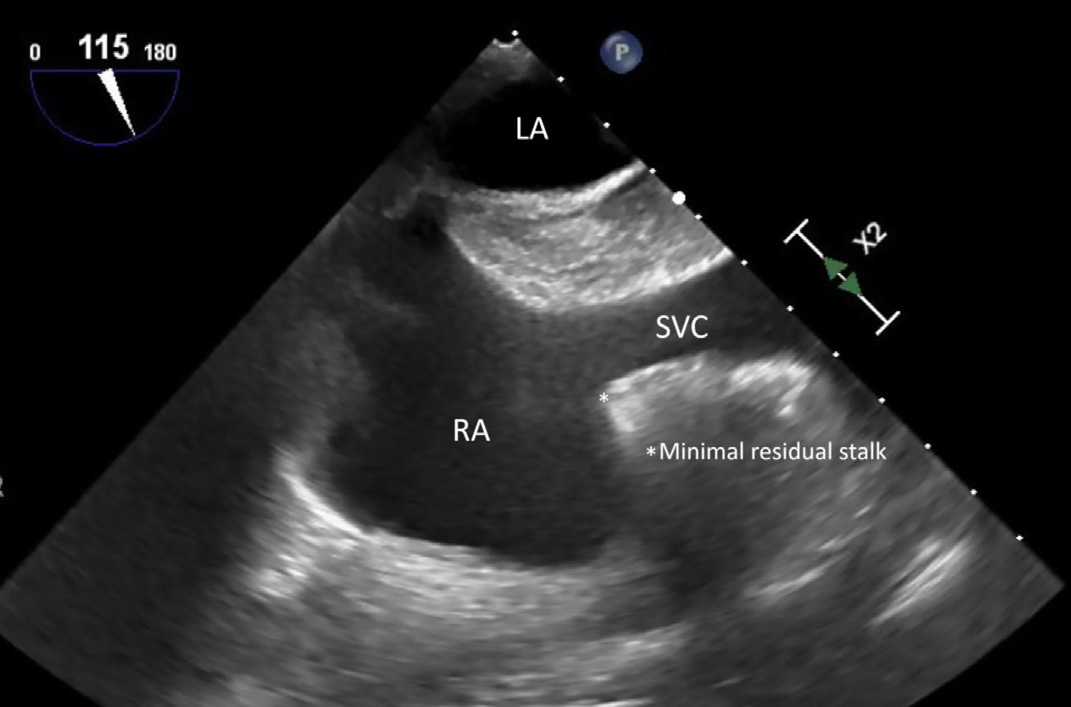
Transesophageal Echocardiography Showing the Residual Stalk of the Mass Attached to the Superior Vena Cava–Right Atrial Junction LA = left atrium; RA = right atrium; SVC = superior vena cava.

She underwent percutaneous aspiration using AngioVac (AngioDynamics). The procedure was performed under general anesthesia. The device is composed of a large-bore inflow cannula that was inserted through the right internal jugular vein. Once approximated to the mass, blood is aspirated—driven by a cardiopulmonary bypass motor—and filtered before being returned to the body through a common femoral venous outflow cannula. The device is a closed circuit that allows continuous suction with adjusted rounds per minute to prevent suction of nonpathological structures. Approximately 94% of the mass was debulked (residual size 0.8 × 0.5 cm) (Figure 3) and the aspirated material was sent to pathological evaluation that showed calretinin, cluster of differentiation 31, cytokeratin AE1/AE3, and cytokeratin 7 positive; cytokeratin 20, GATA-binding protein 3, and thyroid transcription factor-1 negative; cluster of differentiation 68 stained few scattered macrophages. Overall consistent with myxoma.

**Figure 4:**
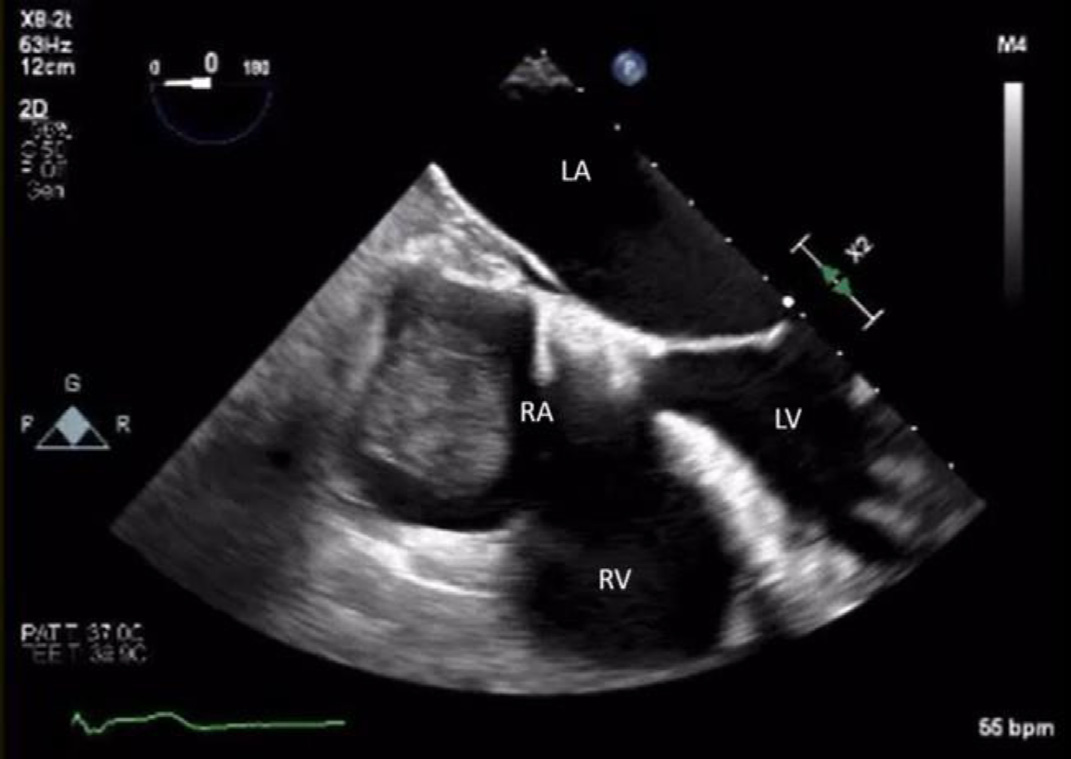
Transesophageal Echocardiography Showing the Residual Stalk of the Mass Attached to the Superior Vena Cava–Right Atrial Junction 3.3 Years Later LA = left atrium; LV = left ventricle; RA = right atrium; RV = right ventricle.

**Figure 5:**
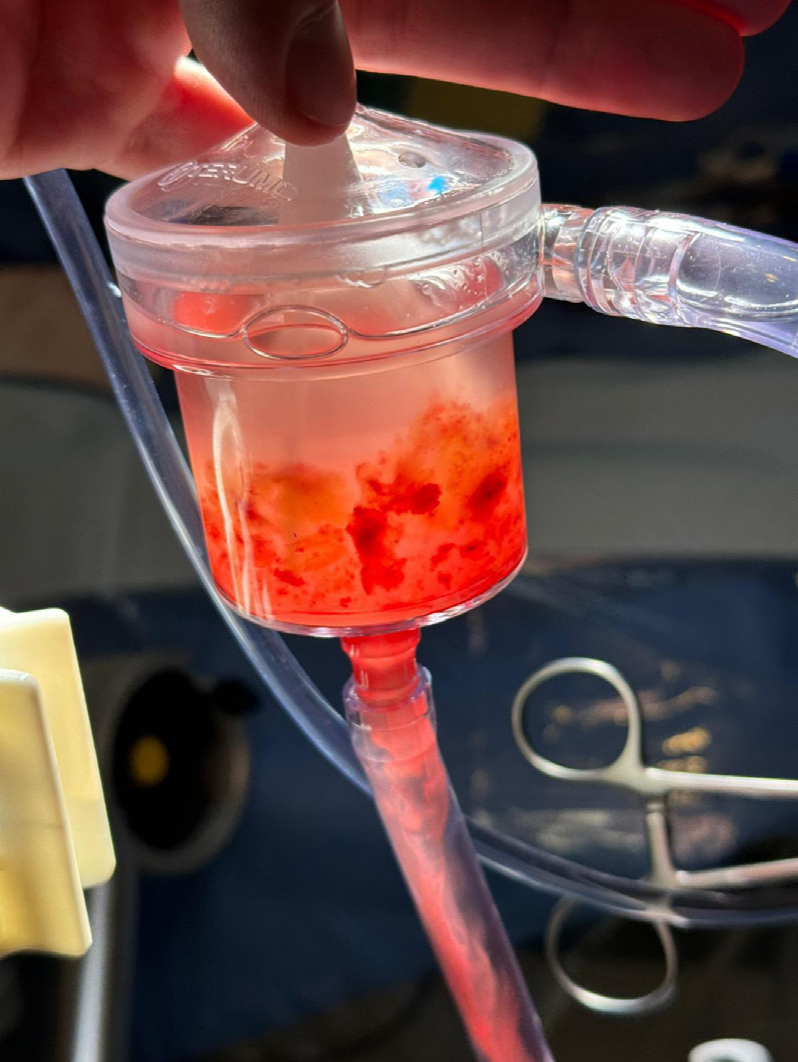
Aspirated Material From the Myxoma in the AngioVac Filter From the Second Procedure

**Figure 6:**
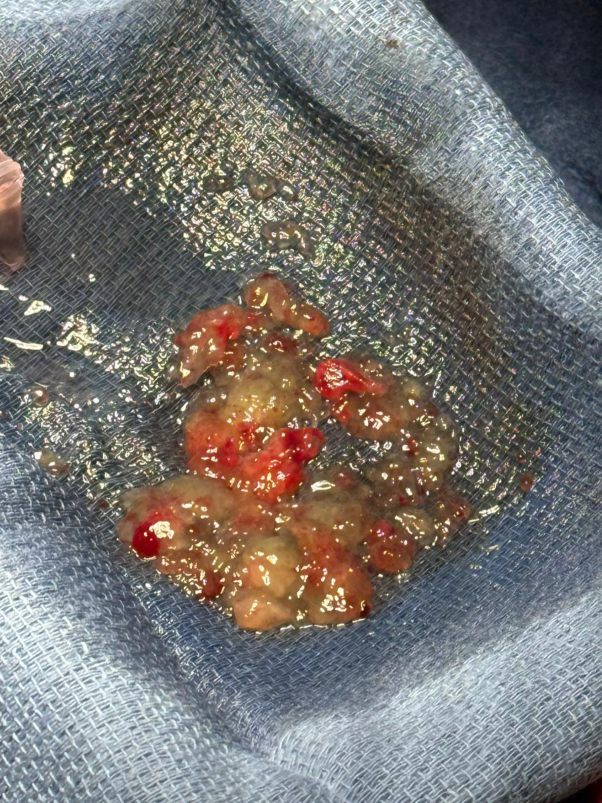
Filtered Material From Percutaneous Aspiration of the Myxoma From the Second Aspiration Procedure

**Figure 7:**
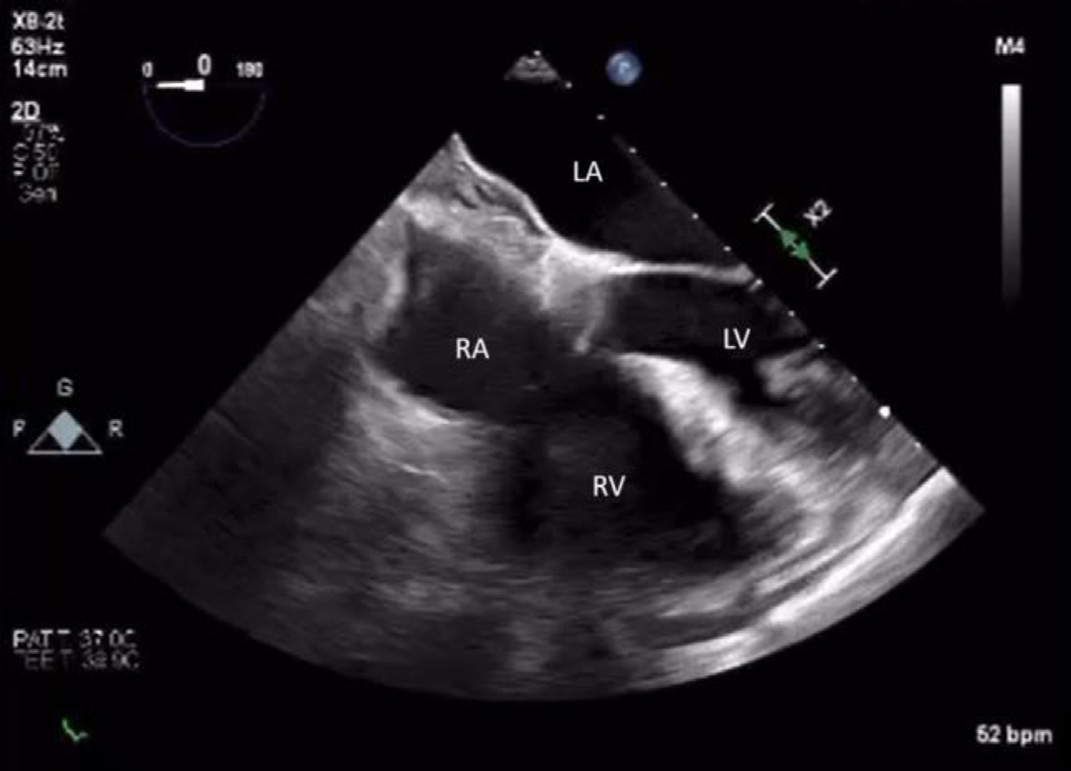
Transesophageal Echocardiography Showing a Thickened Intra-Atrial Septum With Minimal Residual Tumor in the Right Atrium After the Second Aspiration Procedure LA = left atrium; LV = left ventricle; RA = right atrium; RV = right ventricle.

The patient was followed with annual echocardiography by her primary cardiologist. Due to continued tumor growth, she was referred to consideration of repeat percutaneous aspiration 3.3 years later. Transesophageal echocardiogram showed (Figure 4) a 3.3 × 2.6-cm mass in the same position. After a multidisciplinary discussion, repeat percutaneous aspiration was pursued to prevent emboli and obstruction of blood flow. More than 95% of the mass was debulked (Figure 5, Figure 6, Figure 7). On pathological examination there was an aggregate of tan to red brown myxoid tissue fragments and histology consistent with myxoma.

## Discussion

Cardiac myxomas are typically treated surgically, yet the operative mortality rate is between 2% and 6%.^2^ Furthermore, recurrence has been described in up to 15% of patients^3^ (particularly in cases of inadequate resection and in familial inheritance etiologies), most occurring during the first 4 years.^4^ Such recurrence-free survival data warrant exploration of alternative options to surgery. Percutaneous aspiration devices emerged as an expanding option for the treatment of cardiovascular masses. Their application expanded from thrombi and emboli to off-label use in endocarditis and cardiac tumors^5^ with no procedural mortality among patients with cardiac masses in RAPID (Registry of AngioVac Procedures in Detail).^6^ Furthermore, intracardiac echocardiography–guided procedures using moderate or local sedation are feasible.^7^ Percutaneous aspiration may have a residual margin that may be associated with higher chances of recurrence compared with surgical resection. Despite incomplete removal of the mass in the current case, the patient remained asymptomatic for more than 3 years. As mentioned, a recurrence after surgical excision would likely occur during the same time frame.

Data regarding growth rate of cardiac myxomas are scarce, showing a range of no progress, (ie, a stable tumor) to a growth rate of 1.36 cm/mo.^8^ To our knowledge, this is the first report to describe the regrowth rate of a myxoma after percutaneous aspiration. The growth rate was 0.06 × 0.05 cm/mo on average (7.2 × 6 mm/y).

## Follow-Up

The patient will require continued annual echocardiography evaluations.

## Conclusions

Until recently, surgery was the only option to treat cardiac myxoma. Newer, percutaneous aspiration techniques are particularly helpful in patients at high surgical risk. Moreover, repeat percutaneous aspiration for right atrial myxoma is feasible for nonsurgical candidates. A successfully debulked myxoma does not necessarily cause obstruction early after the initial procedure, thereby supporting a possibly sustainable novel mid-term solution for selected patients. The regrowth rate was comparable to that of in situ tumors, suggesting predictability with follow-up. More experience is required for confirmation of the safety of this approach and to better define the range of regrowth rate.

## Funding Support and Author Disclosures

Dr Rozenbaum has received consultant fees from Angiodynamics and Dr Gnall has received advisory fees from Livanova, ABIOMED, and Maquet. The open-access fees for the current study are funded by Angiodynamics.
